# Synthesis and Characterization of Blended Cellulose Acetate Membranes

**DOI:** 10.3390/polym14010004

**Published:** 2021-12-21

**Authors:** Abdullah M. Asiri, Francesco Petrosino, Valerio Pugliese, Sher Bahadar Khan, Khalid Ahmad Alamry, Soliman Y. Alfifi, Hadi M. Marwani, Maha M. Alotaibi, Catia Algieri, Sudip Chakraborty

**Affiliations:** 1 Chemistry Department, Faculty of Science, King Abdulaziz University, Jeddah 21589, Saudi Arabia; sbkhan@kau.edu.sa (S.B.K.); kbadahdah@kau.edu.sa (K.A.A.); Salfaifi@kau.edu.sa (S.Y.A.); zsudip.c14@gmail.com (H.M.M.); vut.vutu@gmail.com (M.M.A.); 2 Department of Computer Engineering, Modeling, Electronics and Systems (D.I.M.E.S.), University of Calabria, Via-P. Bucci, Cubo-42A, 87036 Rende, CS, Italy; f.petrosino@dimes.unical.it (F.P.); valeriopugliese@libero.it (V.P.); 3 Institute on Membrane Technology, ITM-CNR, Ponte P. Bucci, Cubo 17/C, 87036 Rende, CS, Italy; c.algieri@itm.cnr.it

**Keywords:** polymeric membrane, photocatalyst, ZnO, cellulose acetate, nano-composite membrane, environmental application

## Abstract

The casting and preparation of ultrafiltration ZnO modified cellulose acetate membrane (CA/ZnO) were investigated in this work. CA membranes were fabricated by phase inversion using dimethylformamide (DMF) as a solvent and ZnO as nanostructures materials. Ultrafiltration (UF) performance, mechanical stability, morphology, contact angle, and porosity were evaluated on both CA- and ZnO-modified CA samples. Scanning electron microscopy (SEM) was used to determine the morphology of the membranes, showing different pore sizes either on rough surfaces and cross-sections of the samples, an asymmetric structure and ultra-scale pores with an average pore radius 0.0261 to 0.045 µm. Contact angle measurements showed the highest hydrophobicity values for the samples with no ZnO addition, ranging between 48° and 82.7° on their airside. The permeability values decreased with the increasing CA concentration in the casting solution, as expected; however, ZnO-modified membranes produced lower flux than the pure CA ones. Nevertheless, ZnO modified CA membranes have higher surface pore size, pore density and porosity, and improved surface hydrophilicity compared with pure CA membranes. The results indicated that the incorporated nano-ZnO tends to limit the packing of the polymer chains onto the membrane structure while showing antifouling properties leading to better hydrophilicity and permeation with consistent UF applications.

## 1. Introduction

Membrane technology has become increasingly important in the recent past, showing considerable advantages over well-known processes, such as adsorption, distillation, and extraction [1,2]. Cellulose acetate (CA) membranes are widely used as fundamental separation instruments in laboratory and industrial processes. Cellulose acetate represents a class of polymers well known in the field of membrane production due to the rapid and economical production method. It is also characterized by interesting properties such as high solubility in polar organic solvents, toughness, and impact resistance; it is stable at room temperature and has good resistance to discoloration when exposed to sunlight. Cellulose acetate (CA) was one of the first membrane polymers that has been used for aqueous-based separations and used as both reverse osmosis (RO) and ultrafiltration (UF) membranes [3].

CA membranes have unique advantages such as their hydrophilicity which supports a considerable volumetric flux and a relatively low propensity to fouling, combined with good mechanical resistance. There are some significant limitations of use since cellulose acetate is not the most suitable polymer for more aggressive removal and has low oxidation and chemical resistance [3,4,5].

Evident improvements in permeability, hydrophilic properties, and the fouling of polymeric membranes has been achieved by blending membranes with polymers or organic compounds using the phase inversion method [6]. The necessity of using CA membranes in processes with gradually different macromolecular components demands the manipulation of the membrane through the interaction and addition of a balanced moiety such as polyacrylonitrile (PAN) with polyvinylchloride (PVC), cellulose acetate (CA) and polysulfone (PSf) [3]. Therefore, combinations and blends of different polymers could represent an attractive solution to improve some specific characteristics of a membrane, such as hydrophobicity and hydrophilicity. [7,8]. Past studies have shown how pairing polymers and nanoparticles have represented an effective method to enhance the membrane hydrophilicity of CA membranes [7] and to contrast the problems related to fouling that cause an immediate decrease in the flow of UF membranes with a consequent increase in operating and maintenance costs. Considering that most foulants are intrinsically hydrophobic, enhancing the hydrophilicity of the membrane can mitigate the fouling [9].

Because of its outstanding qualities, cellulose acetate (CA) has been extensively used in various unit operations, such as UF, adsorption, and rejection.

A summary of different commercial membranes blended with additives is reported in Table 1 [10].

Numerous procedures are practiced to reduce membrane fouling. Significantly, the blend of polymer and nanoparticles was proven successful in enhancing membrane hydrophilicity. As mentioned in many works, TiO_2_ nanocomposite-based polymeric membranes were extensively studied in the past [11,12]. In the meantime, in order to find a viable alternative, several articles have proved that zinc oxide (ZnO) nanoparticles, more economical than the cost of TiO_2_, exhibited comparable physical and chemical characteristics with TiO_2_ [13].

Hence, ZnO nanoparticles are considered an adequate competitive substitute to TiO_2_ nanoparticles in developing antifouling organic–inorganic composite membranes [12]. ZnO has shown the characteristics of an excellent nanomaterial, and it has been extensively used for photocatalysis and antifouling purposes; indeed, it retains strong hydrophilicity by adhering to the hydrophilic functional groups such as –OH, –SO_3_H, and –COOH [14,15]. Moreover, an essential benefit of employing ZnO is the possibility of bonding the nanoparticle oxide on the membrane surface through the chemical reaction between the oxide and the polymeric membrane [12].

## 2. Materials and Methods

### 2.1. Materials

Cellulose acetate (CA; average acetyl content of 39.7 wt%, average MW = 50,000 (GPC)), (Sigma Aldrich, Burlington, MA, USA), is used as a polymer component for membrane preparation. The N,N-dimethylformamide (DMF) with an analytical purity of 99% (Sigma Aldrich, Burlington, MA, USA) is used as a solvent during polymer blending. Zinc oxide (ZnO, 50 nm) (Merck, New York, NY, USA) is employed as a membrane additive. A glycerol (HHoneywell, Morristown, NJ, USA) solution was also used in the membrane washing operations.

### 2.2. Membrane Preparation

The different membranes are prepared by the phase inversion method that permits the preparation of asymmetric and symmetric membranes [16,17]. In this method, a thermodynamically stable polymeric solution, by means of a demixing process, passes from liquid to solid state in a controlled manner. In particular, the demixing process can be obtained by using a non-solvent (NIPS or non-solvent induced phase separation). In the NIPS, a stable polymeric solution is casted on a support in a thin film. Subsequently, the support is immersed in a coagulation bath containing a non-solvent (usually water). The exchange between the solvent and the non-solvent induces a phase separation which leads to the formation of a porous structure [18].

In this work, the polymeric solution, obtained by dissolving the CA in DMF, is stirred at 75 °C (by using a water bath) for 3–4 h to ensure a complete polymer dissolution into the solvent as also reported in the open literature [19]. Subsequently, the polymeric solution is left at 60 °C for 8 h. Then, the polymer solution is kept in the fume hood for around 12 h to remove the air bubbles, the polymeric solution is poured on a glass surface at room temperature by using a casting knife (Elcometer-3450). The glass plate is kept at room temperature for a few minutes and then it is immersed in the coagulation bath to initiate the phase inversion. In each replication of membrane fabrication, the environmental parameters as well as the solution viscosity is kept constant for a uniform membrane porosity.

The blended membranes are obtained with the addition to the CA solutions of a certain amount of ZnO nanoparticles. To avoid the formation of aggregates/clusters, the solution is kept in an ultrasonic bath at 60 °C for 8 h [19]. The degassed solution is casted on a glass plate using a casting knife (Elcometer 3530/5) and a 150 mm casting gap described elsewhere [20]. The casted membranes are immediately immersed into a coagulation bath for 1 h. The precipitated membrane is taken out of the coagulation bath and rinsed with running distilled water to remove the excess solvent. At last, the wetting membrane is dried in air at room temperature until a dry flat-sheet porous membrane is prepared. The dried membrane is then immersed into a 10% glycerol in water solution for preservation. The different composition of the prepared membranes is reported in Table 2. The composition of all suspensions is expressed by considering a weight percentage (wt%) of the ZnO with respect to the total solute mass.

#### 2.2.1. Experimental

The physical characteristics of the membranes were assessed using a combination of techniques, including scanning electron microscopy (SEM), contact angle (CA), X-ray diffraction (XRD), and thermal analysis (TA). TGA analysis of the blended membrane samples was completed by applying an SDT-Q600 thermogravimetric analyzer at a rate of 10 °C/min. The thermograms for TGA were obtained using a cycle of heating from an ambient temperature to 600 °C. To remove all caustic gases during degradation, a flow of N_2_ gas was maintained at 15 mL/min.

#### 2.2.2. Membrane Morphology Characterization

The top view and cross-section of the membrane samples were examined by scanning electron microscopy (SEM), using a Cambridge Zeiss LEO 400 microscope. The membrane cross-section was cut in the appropriate size, after it was fractured in liquid nitrogen to keep unaltered the film structure and coated with gold to reduce the charging effects on the polymer surface.

#### 2.2.3. Membrane Porosity

Membrane pore dimensions were determined through a capillary flow porometer CFP 1500 AEXL (Porous Materials Inc. PMI, Ithaca, NY, USA). A portion of the membrane was thoroughly wetted with Galwick (15.9 dyne/cm) as the wetting liquid, and tests were executed using the wet-up/dry-up method. The measurement of the bubble point, the largest pore size, and pore size distribution was based on Laplace’s equation (Equation (1)) [21]:

Dp = 4γcosθ/P(1)

where dp is the pore size diameter, γ is the surface tension of the liquid, θ is the contact angle of liquid, and P is the external pressure. The membrane was collocated on the sample chamber, and then the chamber was sealed. Pure oxygen was then allowed to flow into the chamber gradually. When the increased oxygen pressure reached a point that overcame the capillary flow of the fluid within the largest pore, the bubble point was found. Following determining the bubble point, the pressure was increased continuously, and the oxygen permeation rate was measured until all pores were empty of Galwick, and the sample was considered dry.

#### 2.2.4. Fourier Transform Infrared (FTIR) Spectroscopy

FTIR spectra of blend membranes were scanned using a Shimadzu IR Prestige-21 instrument equipped with a variety of sampling accessories, including horizontal attenuated total reflectance (HATR) accessories. The air background of the instrument was run before each sample. The frequency range was from 4000–600 cm^−1^ at 60 scans per spectrum.

#### 2.2.5. Membrane Physical Property Characterization

Tensile testing of pure and blended membranes was determined at room temperature using a tensile testing machine (Zwick/Roell Proline Z005 equipped by a Load Cell Xforce P). The experiments were performed with a crosshead speed of 3 mm/min. The sample width was 25 mm and the length between the jaws was 45 mm.

#### 2.2.6. Surface Hydrophilicity and Contact Angle Measurement

Water contact angles were measured using the tensile drop method at an ambient temperature by using a CAM 200 contact angle meter (KSV Instruments LTD, Helsinki, Finland), depositing an ultrapure water drop (5 µL) on the membrane surface by means of an automatic micro-syringe. For all membranes, at least 4 measurements were taken and the average value and the corresponding standard deviation were then calculated.

#### 2.2.7. XRD Analysis

To confirm the presence of nanoparticles in the membranes, the X-ray diffraction analysis was performed using Bruker equipment (D8 ADVANCE axes) with a monochromatic Cu Kα radiation (λ = 0.154 nm) source, operated at 40 mA and 40 kV between 20° and 70°. Finally, thermal analysis of the membranes was evaluated using a simultaneous thermal analyzer (Netzsch, STA429 CD) under a nitrogen atmosphere at a heating rate of 10 °C/min. from 20 °C to 800 °C.

#### 2.2.8. Pure Water Permeability

**Figure 1:**
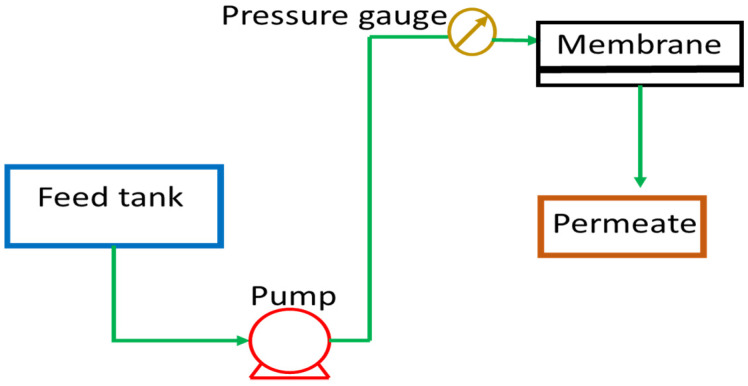
Scheme of the ultrafiltration lab-scale plant.

Pure water was used for testing the permeation properties of the different prepared membranes. The scheme of the dead-end ultrafiltration (UF) experimental apparatus is showed in Figure 1.

The permeate flux was evaluated by using the following equation (Equation (2)):

J = V/(A × t)(2)

where J is the permeate flux (L/m^2^h), V is the volume of the accumulated permeate, A is the membrane surface area and the filtration time. The effective membrane surface area was equal to 12.68 cm^2^. For each experiment, three different transmembrane pressures were analyzed, and every experiment was carried out at least in triplicate.

#### 2.2.9. Zeta Potentials

Membrane zeta potentials were measured on fabricated membranes using streaming potential performed with the adjustable gap cell in the SurPASS system provided by Anton Paar, Austria. The zeta potential ζ was then calculated by following equation (Equation (3)). It is the average of at least three different samples analyzed.

(3) $$\zeta =\frac{dU}{dp}\frac{\eta }{\varepsilon \varepsilon_{o}}k$$

In the above equation (Equation (3)) *U* is the streaming potential, *p* the pressure, *η* is the viscosity of the electrolyte solution, ε is the dielectric constant of the electrolyte solution, *ε_0_* is the vacuum permittivity and k is the electrolyte conductivity.

## 3. Result and Discussion

### 3.1. SEM Analysis of the Membrane Surfaces

**Figure 2:**
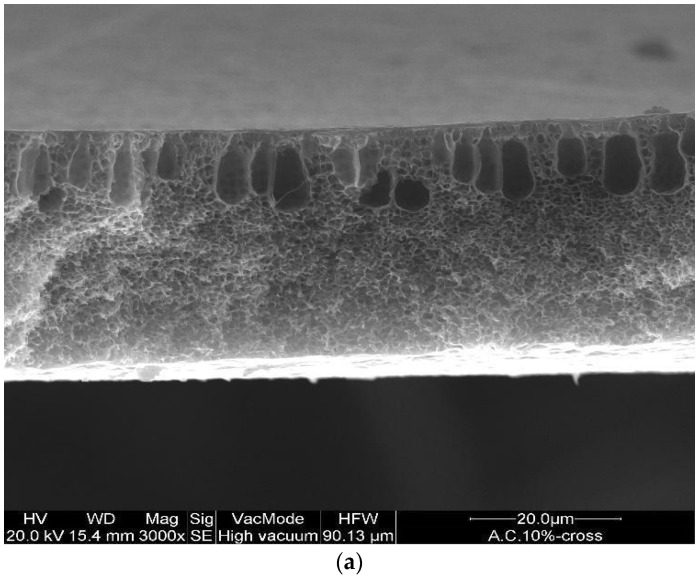

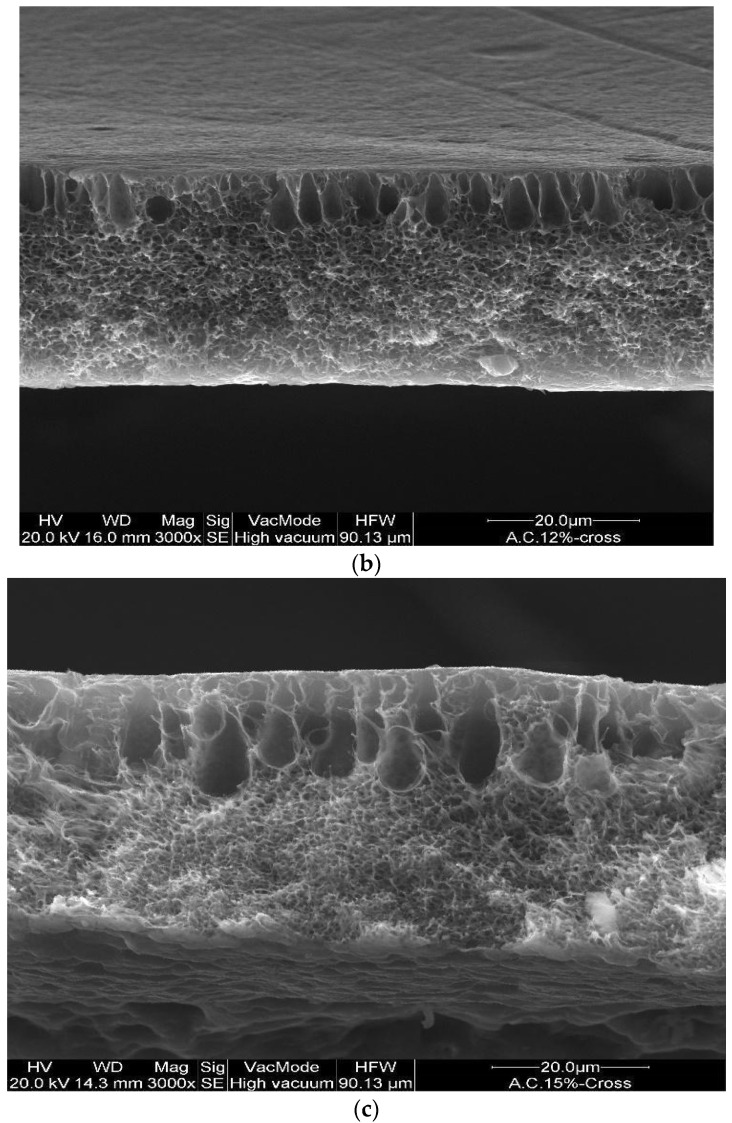
Cross-section of the asymmetric CA membranes at different polymer concentration: (**a**) 10 wt% (**b**) 12 wt% and (**c**) 15 wt%.

The effect of polymer concentration on the morphological properties of asymmetric membranes was evaluated by SEM analyses. The membranes prepared at different polymer concentrations exhibited an asymmetric cross-section structure, with a thin selective layer, and finger-like and sponge-like pore structures in the sub-layer (see Figure 2a–c). The formation of the skin layer is due to the instantaneous demixing of solvent and non-solvent during the phase inversion process [22]. This structure is due to the high mutual diffusivities of water and DMF during the phase separation [23]. In addition, the formation of macro-voids is obtained when the diffusion rate of the non-solvent into the polymer-poor phase overcomes the solvent diffusion rate. An increase in the polymer concentration determined a decrease in the dense skin layer. This result is attributed to the higher viscosity of the dope solution that blocks the diffusion exchange between solvent (DMF) and non-solvent (water).

**Figure 3:**
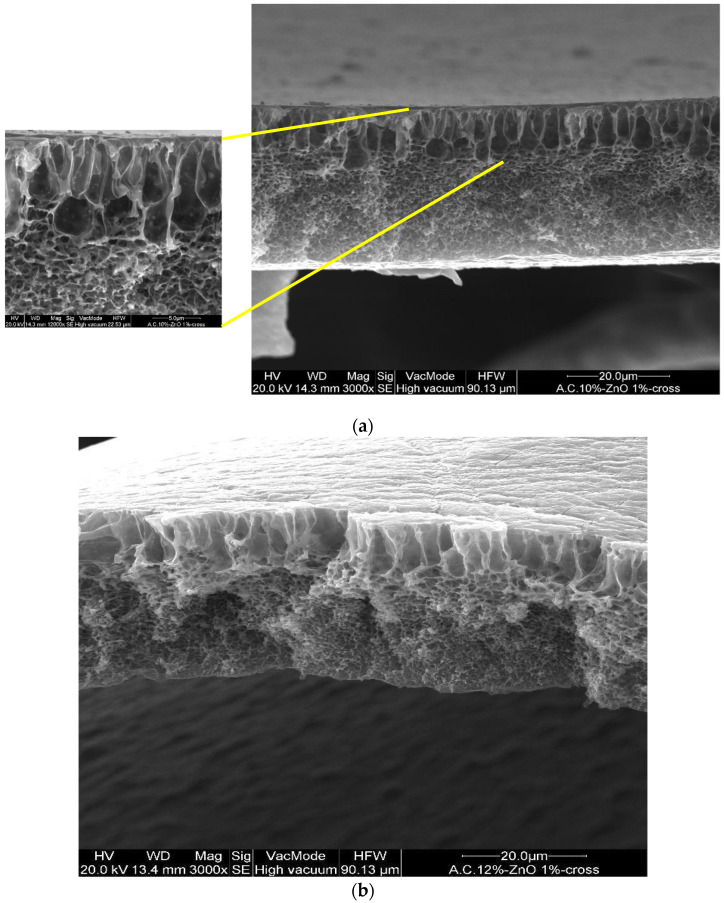

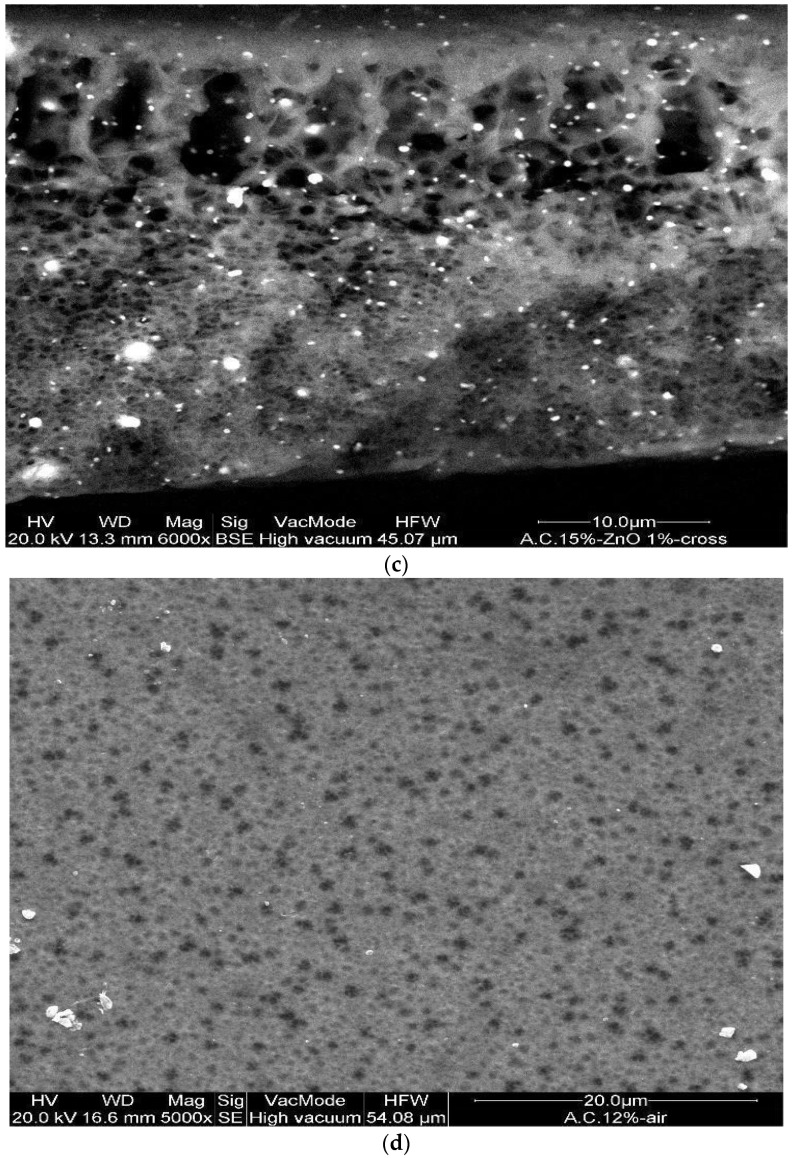

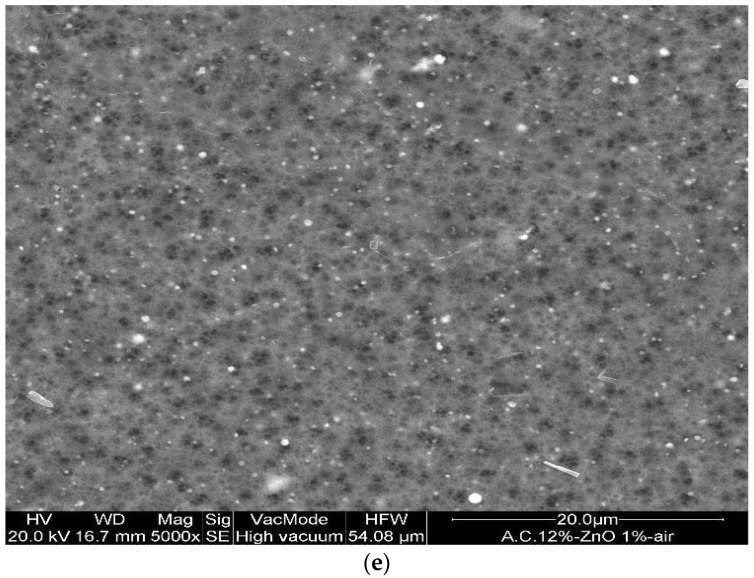
Cross-section of the asymmetric blended membranes: (**a**) CA10%–ZnO1%; (**b**) CA12%–ZnO1%; (**c**) CA15%–ZnO1% (this last image has been acquired in BSE). Top view of the membranes: (**d**) CA12% and (**e**) Ca12%–ZnO1%.

In addition, the CA–ZnO membranes, prepared at different inorganic nanoparticle concentrations, showed an asymmetric structure similar to the bare polymeric ones; in Figure 3a,b illustrates the cross-section of different fabricated membranes with different concentrations of CA as well as of ZnO. However, the addition of the ZnO determined an increase in the pore amount because its addition strongly influences the thermodynamic and kinetic factors during the membrane formation prepared by the phase inversion technique [12]. In fact, the addition of the inorganic nanoparticles caused an increase in suspension viscosity, and so the diffusion of the non-solvent decreased, and so the formation of macro-voids was suppressed and so a higher number of pores were formed in the skin layer. A comparison between the top view of the bare and blended membranes evidenced what was previously reported (see Figure 3d,e).

### 3.2. FTIR Analysis of Blended Membrane

**Figure 4:**
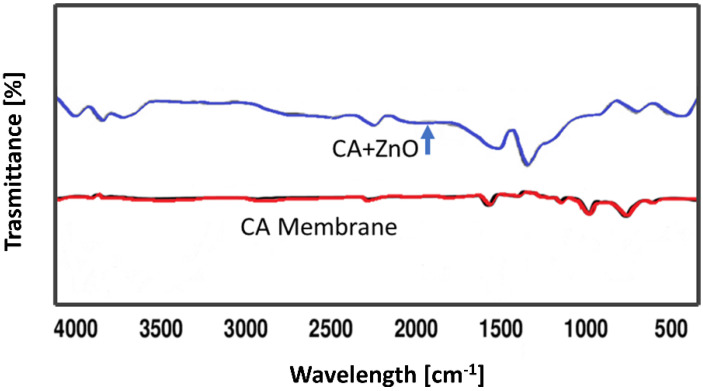
FTIR spectra of CA and CA-ZnO blended membrane.

FTIR spectra of the virgine CA as well as CA–ZnO blend membrane were performed to understand the interactions between intermoleculs of CA as well as blend the CA/ZnO membrane (Figure 4). In the following FTIR spectrum of the virgine CA membrane, a weak broad band between 3600 and 3250 cm^−1^ is present which represented the stretching vibration of –OH bond, whereas the strong band around 2922.16 and 1369.46 cm^−1^ is assigned to the aliphatic stretching of C–H vibration. The stretching modes due to C=O were located at 1739.79 cm^−1^ while by C–O–C at 1222.87, 1033.85 cm^−1^. The band obtained at 902.69 cm^−1^ was attributed to a pyranose ring present in cellulose acetate as described elsewhere before.

On the other hand, in the other FTIR spectrum of virgine CA/ZnO blended membrane, the broad band at 3617 cm^−1^ represented the stretching vibration of O–H. The strong peak around 2317 cm^−1^ was assigned to the stretching vibration of the carboxylic acid hydroxyl group, and another strong peak around 1520.65 cm^−1^ was attributed to the stretching mode of the C=C bond. The characteristic peak around 710.83 cm^−1^ represented the out-of-plane bending of the C–H vibration. The strong peaks around 3602.18 cm^−1^, 3393.43–3379.79 cm^−1^, 2913.55–2903.31 cm^−1^ and 2436.31–2419.64 cm^−1^ in the FTIR spectrums of blend membranes were attributed to the stretching vibration of O–H. The bonds due to C≡C, and C=C stretching mode were located around 2133.64–2127.23 cm^−1^, and 1526.57–1524.93 cm^−1^. The characteristic peaks around 725–721 cm^−1^ represented the out-of plane bending of C–H vibration. It can be concluded that the presence of such O–H· · ·C=O interaction indicated an exceptional mixing of CA and ZnO in the blend membranes. It is also observed that in the spectra of the CA/ZnO blend membranes, broad band at around 1688.43–1681.81 cm^−1^ represented the stretching vibration of C=N due to the presence of a DMF ring, compared with the spectrum of the CA membrane.

### 3.3. Mechanical Property

**Figure 5:**
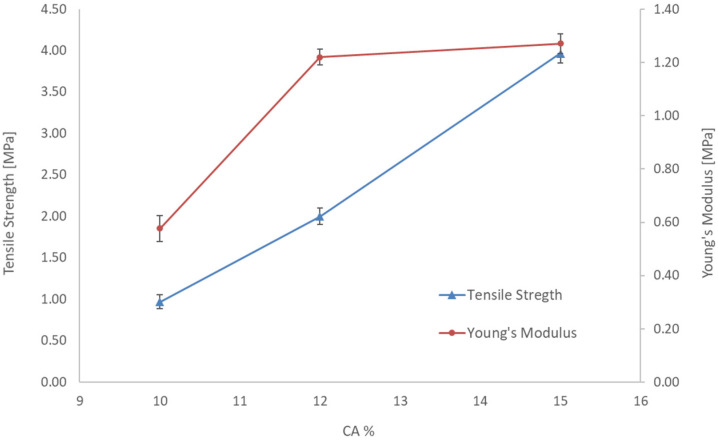
Tensile strength and Young’s modulus of the CA membranes prepared at different polymer concentrations.

The effect of the polymer concentration on the mechanical property of the membranes was also evaluated by measuring the tensile strength and the results are illustrated in Figure 5. The standard deviation resulted as less than 5%. In particular, the tensile strength increased with the polymeric concentration due to the higher porosity and the stiffening of the membrane structure with the polymer concentration [24].

**Figure 6:**
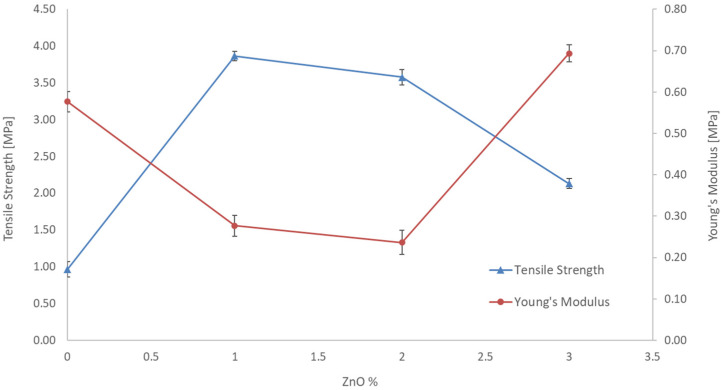
Tensile strength and Young’s modulus of the blended membranes prepared with a CA concentration of 10 wt%.

In order to investigate the effect of ZnO in Figure 5, tensile strength and Young’s modulus of membranes n. 1, 2, 3 and 4 are reported. For a fixed content in CA (10%) and different amounts of ZnO the Figure 6 shows a decrease in Young’s modulus and an increase in tensile strength for the membrane with 1 and 2% of ZnO. The standard deviation resulted as less than 5%. This result was derived from the incorporation of ZnO additive could be due to the increased flexibility of the CA chains caused by a good interaction between the filler particles (ZnO) and the polymeric chains of CA and consequently transferred the stress between fillers and polymer, thus showing a lower modulus and higher strength. However, with a higher percentage of ZnO (3%) the filler polymer interactions may decrease, and an opposite effect is observed with higher Young’s modulus but lower strength.

The mechanical stability of the blended membranes increased with the ZnO concentration up to 1 wt% of ZnO. However, increasing the ZnO content further was observed as a weakening of the mechanical stability. These experimental data could be explained by considering the suppression of the macro-void, the increase of viscosity of casting suspension and the weak chemical interaction between inorganic filler and polymeric chains. However, an excessive filler concentration determined the nanoparticle cluster formation that has led to weakening of the mechanical stability. The interaction between inorganic filler and polymers resulted in the increase in the mechanical strength of the membrane. However, an excessive filler concentration can cause the nanoparticles aggregation and make them not able to disperse uniformly in the polymeric matrix, which forms many stress convergence points in the membrane system under the action of outside force.

### 3.4. Contact Angle Measurement

The water contact angle measurements of the pure CA membranes demonstrated a moderate surface wettability (highest contact angle value). The addition of ZnO nanoparticles enhanced the surface hydrophilicity of the membranes as also reported in the open literature [25]. The membranes become more hydrophilic with the ZnO nanoparticles addition. In particular, ZnO nanoparticles embedded in the membrane surface are capable of forming hydrogen bonds with water molecules, resulting in the increased adsorption capacity of water and improved hydrophilicity of the membrane [26,27]. The contact angle values are reported in Table 3.

### 3.5. XRD Analysis of the Membrane Surfaces

**Figure 7:**
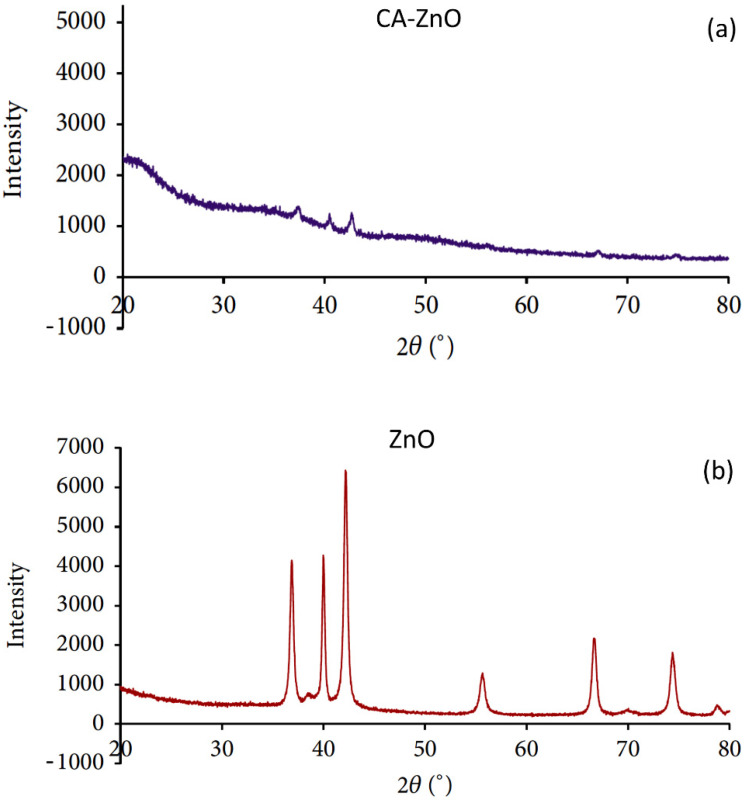
XRD patterns of (**a**) CA- ZnO membrane and (**b**) ZnO nanoparticles.

The presence of ZnO nanoparticles in the membrane matrix is confirmed by XRD analysis. Figure 7a,b show the diffractograms of CA supported ZnO nanoparticles: (A) membrane, and ZnO nanoparticles, and (B) the ZnO diffractogram exhibited dominant peaks at 2θ angles of 36.91°, 39.98°, 42.17°, and 55.78° which correspond to the main characteristic peaks of zinc-oxide nanoparticles. The CA’s composite membrane also showed the characteristic peaks of ZnO nanoparticles The XRD results indicated the presence of ZnO nanoparticles in the composite membranes.

### 3.6. Thermal Analysis of the Membranes

**Figure 8:**
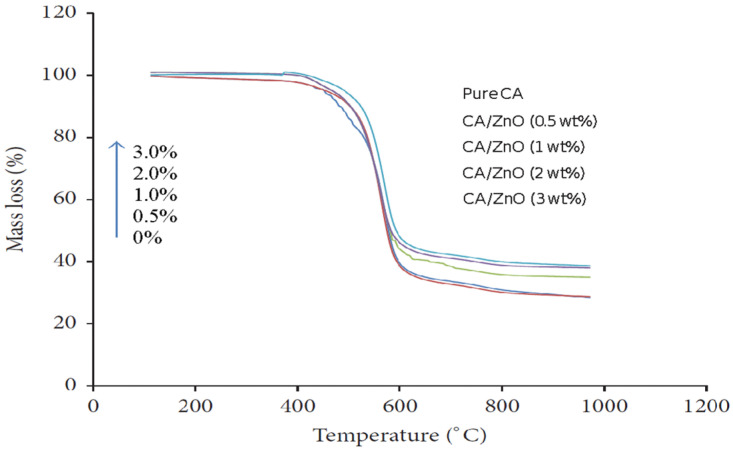
TGA results for increasing decomposition temperature for (0.0, 0.5, 1.0, 1.5, and 2.0 wt%) of ZnO on CA membranes.

Thermal analysis (TGA) provides information on the decomposition temperature (Td) of a substance. It is defined as the temperature at 3% weight loss. A simultaneous thermal analyzer (Netzsch, STA429 CD) was used to investigate the weight loss of both the bare CA and composite ZnO membranes. The decomposition temperature of the pure CA and CA/ZnO membrane followed a similar trend. The temperature gradually increased with the increasing addition of ZnO on CA membranes (Figure 8). Addition of ZnO nanoparticles improved the thermal stability of the CA membranes.

In ultrafiltration experiments, the effect of the polymer concentration and ZnO addition on the permeability was considered. Below, Table 4 shows the permeability values of the prepared membranes. The permeability of the CA membranes decreased with increased polymer content in the dope solution owing to the reduction of the macro-void formation [28,29,30]. The blended membranes displayed an increase in the permeability explained by considering the coupled action of improved hydrophilicity and the increased membrane porosity. In addition, the membranes are prepared at a CA concentration of 15 wt% did not permeate in the investigated range of transmembrane pressure.

### 3.7. Zeta Potential of Blended Membranes

The zeta potential measurements were carried out by using sodium chloride solutions at different concentrations (0.001 M, 0.01 M, 0.05 M, and 0.1 M) at various salt concentrations of salt solution. The highest zeta potential was found for the CA–ZnO membranes (zeta potential = +23 mV at pH 7 and 0.01 M sodium chloride). These results indicate the possibility to reject chemical species with opposite zeta potential value. These results indicate as the CA–ZnO membranes exhibited an in of the antifouling ability.

## 4. Comparison with Other Flat-Sheet Membranes Prepared

Table 3 lists a performance comparison between the different membranes performed in this current work. The operating conditions used for each experiment were room temperature, three different transmembrane pressures (1.5, 2.5 and 3.5 bar) and every experiment was performed at least in triplicate [31,32].

The prepared flat-sheet composite membrane exhibited comparable or even better performance than commercially available flat-sheet membranes. In fact, the permeate flux of the prepared membranes was comparable or higher to that of the commercial membranes or even higher than in some cases. Moreover, to analyze the behavior of a backwashing cycle, the same membranes were used for a filtration cycle of a 0.1 g/L BSA solution and then were subjected to a back-washing cycle with distilled water at 3 bar. After that, the permeability experiments were carried out again and the values were found to be within a range of 10% of the previous results reported in Table 3. The regenerated membranes were used again for the test with a small decrease in permeate flux.

However, it should be noted that this study is one of the first works performed with ZnO to fabricate a composite membrane to study its different physio-chemical properties in order to choose the best possible membrane for further research work. It is observed that there was no “blind pore” in the membrane and the porosity was also higher than that of only CA membrane prepared through phase inversion process. The mechanical strength of the CA blended ZnO membrane was very poor compared with that of the prepared flat-sheet composite membrane to 2% of ZnO. It is believed that if the membrane preparation optimizations which were attempted in this work are accompanied with a higher concentration of CA, and optimized amount of ZnO, higher flow rate, and flux will be obtained and more efficient module designs can be performed to improve the flow pattern and diminish the polarization effect, and an even higher flux is achievable in the photocatalysis process.

## 5. Conclusions

In this work, ultrafiltration ZnO modified cellulose acetate membranes (CA/ZnO) were prepared by the phase inversion method. The addition of different amounts of ZnO and polymer heavily affected membranes’ morphologies and properties, enhancing surface pore size, contact angle, porosity, and surface hydrophilicity. In particular, a comparative study between CA pristine and CA/ZnO membranes was also performed to analyze the concrete contribution of the nanoparticles on the membrane properties.

The ultrafiltration tests evidenced that the permeability of the CA membranes decreased with the polymer content. Meanwhile, the blended membranes exhibited an increase in permeability due to the coupled effect of improved hydrophilicity and membrane porosity. However, a high number of nanoparticles determined the formation of clusters which are not uniformly dispersed in the polymeric matrix, and so many stress convergence points in the membrane system are formed under the action of outside force. It is possible to observe that a small amount of supplemented nano-ZnO was sufficient to increase the mechanical properties, particularly with the filler concentration of up to 1 wt% of ZnO. The addition of ZnO nanoparticles enhanced the surface hydrophilicity due to the network of hydrogen bonds between CA and ZnO nanoparticles promoting adequate wettability. Optimizing the ZnO/CA ratio in membrane casting could represent a solid starting point for future UF applications to reach compaction resistance, increase mechanical and chemical properties and obtain higher flow rate and flux.

## Figures and Tables

**Table 1:** Summary of cellulose acetate composite membranes.

Polymer Type	Additives	Composition Type	Unit Operation	Application
Cellulose acetate/PAN	Ag nanoparticles	Thin film composites	Filtration/antimicrobial	Salt rejection/Anti-biofouling
Cellulose acetate/Cellulose triacetate	Boehmite	Mixed matrix	Filtration	Salt rejection
Cellulose acetate	SDS	Thin film composites	Filtration	Rejection of pesticides
	L-dopa	Thin film composites	Filtration	Antifouling
	Alkyl derivative of resorcinarene	Polymer Inclusion	Adsorption/filtration	Removal of Pb (II), Cd (II), and Zn (II)
	Iron nanoparticles	Mixed matrix	Filtration	Rejection of phosphates and organic pollutants
Cellulose acetate/PANI	Phytic acid	Mixed matrix	Adsorbent	Removal of Hg(II) and Cr(VI)
Cellulose acetate/PEG	SiO_2_	Mixed matrix	Filtration	Salt rejection
Cellulose acetate/PEG-600	Ag	Mixed matrix	Antimicrobial	Salt rejection/Anti-biofouling

**Table 2:** Membrane composition.

Sample	Cellulose (%)	ZnO (%)	DMF (%)	Cellulose (g)	DMF (g)	ZnO (g)
CA10	10	0	90	2	18	0
CA10-Z1	10	1	89	2	17.8	0.2
	10	2	88	2	17.6	0.4
CA10-Z3	10	3	87	2	17.4	0.6
CA12	12	0	88	2.4	17.6	0
CA12-Z1	12	1	87	2.4	17.4	0.2
CA12-Z2	12	2	86	2.4	17.2	0.4
CA12-Z3	12	3	85	2.4	17	0.6
CA15	15	0	85	3	17	0
CA15-Z1	15	1	84	3	16.8	0.2
CA15-Z2	15	2	83	3	16.6	0.4
CA15-Z3	15	3	82	3	16.4	0.6
CA18	18	0	82	3.6	16.4	0
CA18-Z1	18	1	81	3.6	16.2	0.2

**Table 3:** Water contact angle measurement.

Membrane Code	Contact Angle
CA (15 wt%)	82.7 ± 2.1
CA (15 wt%)–ZnO(1 wt%)	76.0 ± 2.3
CA (15 wt%)–ZnO(2 wt%)	72.6 ± 0.92
CA (15 wt%)–ZnO(3 wt%)	72.0 ± 1.2
CA (12 wt%)	82.2 ± 0.7
CA (12 wt%)–ZnO(1 wt%)	77.1 ± 2.3
CA (12 wt%)–ZnO(2 wt%)	72.5 ± 2.2
CA (12 wt%)–ZnO(3 wt%)	71.4 ± 1.4
CA (10 wt%)	68 ± 2.6
CA (10 wt%)–ZnO(1 wt%)	62.1 ± 4.0
CA (10 wt%)–ZnO(2 wt%)	58.61 ± 1.6

**Table 4:** Permeability values for the different prepared membranes.

Permeability (LMH/bar)	ZnO 0%	ZnO 1%	ZnO 2%	ZnO 3%
CA 10%	250	660	488	1897
CA 12%	213	457	659	2268
CA 15%	0	0	0	0

## Data Availability

The data that support the findings of this study are available from the corresponding author upon valid reasonable request and pending authorization after patent grant.
